# Efficacy of transconjunctival approach for the treatment of orbital fractures: A protocol for systematic review and meta-analysis

**DOI:** 10.1097/MD.0000000000020536

**Published:** 2020-07-17

**Authors:** Yan-Xiu Qi, Si-ying Li, Dong-lan Wang, Ping-ping Zhou

**Affiliations:** Department of Ophthalmology, First Affiliated Hospital of Jiamusi University, Jiamusi, China.

**Keywords:** efficacy, orbital fracture, safety, transconjunctival approach

## Abstract

**Background:**

This study aims to assess the efficacy of transconjunctival approach (TCA) for the treatment of orbital fractures (OF) comprehensively and systematically.

**Methods:**

In this study, we plan to search electronic databases of Cochrane Library, MEDLINE, EMBASE, Web of Science, Allied and Complementary Medicine Database, Chinese Biomedical Literature Database, China National Knowledge Infrastructure and for relevant randomized controlled trials. All these databases will be searched from inception to the March 1, 2020 without limitations of language and publication status. Two independent authors will carry out study selection, data collection, and study quality assessment. Any disagreements will be resolved by discussion with another author if necessary. The study quality will be assessed using Cochrane risk of bias tool. Statistical analysis will be conducted using RevMan 5.3 software.

**Results:**

This study will be the first 1 to exert direct evidence to evaluate the efficacy of TCA for the treatment of OF.

**Conclusions:**

The findings of this study will provide an exhaustive view of TCA for the treatment of OF.

**Study registration number:**

INPLASY202040154.

## 1 Introduction

Orbital fractures (OF) are among the most common facial fractures among both young and adult population, but more common in adults.^[1–4]^ It is reported that such condition accounts for more than 16% of all facial factures.^[5]^ Repair and reconstruction of OF should be undertaken to restore premorbid orbital contours with the greatest possible precision.^[6–10]^ Surgery schedules are recommended for the treatment of OF, such as transconjunctival approach (TCA).^[11–19]^ To determine if it is the perfect way to treat such disorder, it is important to conduct a systematic review to assess the efficacy and safety of TCA for the treatment of OF. Therefore, this study is carried out to evaluate the efficacy and safety of TCA for the treatment of OF.

## 2 Methods

### 2.1 Study registration

We registered this study in the INPLASY202040154. We have prepared this study according to the statement of Preferred Reporting Items for Systematic Reviews and Meta-Analysis Protocols.^[20]^

### 2.2 Criteria for including studies

#### 2.2.1 Types of studies

We will include randomized controlled trials that focus on assessing the efficacy of TCA for the treatment of OF. However, we will exclude any other studies, such as animal studies, non-clinical trials, non-controlled trials, and non- randomized controlled trials.

#### 2.2.2 Types of interventions

In the experimental group, all patients received TCA for treating patients with OF.

In the control group, all patients received any interventions for the treatment of eligible patients, except TCA.

#### 2.2.3 Types of participants

We will include participants who were diagnosed as OF regardless their country, race, sex, and economic sources.

#### 2.2.4 Types of outcome measurements

Outcome measures are eyeball protrusion, eye movement, diplopia, sensory disturbance in the inferior orbital innervation area, orbital volume, defect area, fat loss, muscle hernia, and complications.

### 2.3 Search strategy

#### 2.3.1 Electronic databases sources

We will search the following databases from inception to the March 1, 2020 without limitations of language and publication status: Cochrane Library, MEDLINE, EMBASE, Web of Science, Allied and Complementary Medicine Database, Chinese Biomedical Literature Database, and China National Knowledge Infrastructure. We will present the search strategy sample of Cochrane Library in Table 1, and will adapt similar search strategies for other electronic databases as well.

**Table 1:**
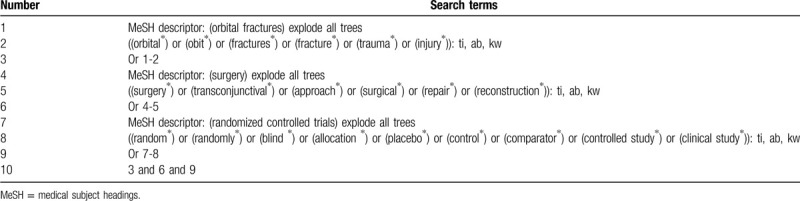
Search strategy of Cochrane library.

#### 2.3.2 Other literature sources

In addition, we will identify grey literatures to avoid missing any potential studies, such as dissertations, ongoing trials from clinical trials registries, conference abstracts, and reference lists of associated reviews.

### 2.4 Study selection

We will export all searched records to the EndNote 7.0, and duplicate records will be removed. Then, we will retrieve the titles and abstracts of all citations and will remove any irrelevant ones. After that, full text papers will be reviewed against all inclusion and exclusion criteria by 2 authors independently. If necessary, any discrepancies between 2 authors will be consulted another author to make a final decision. We will show the results of all study selection in a flowchart.

### 2.5 Data extraction and management

We will extract all essential data from included studies following full text screening using data extraction sheet. Two authors will collect data independently, and any different opinions between 2 authors will be solved by a third author through discussion to reach a consensus decision. The extracted information includes study characteristics (such as title, authors, journal, time of publication, study type, study setting, inclusion and exclusion criteria, et al), patient characteristics (race, age, gender, diagnostic criteria, et al), details of intervention and control, primary and secondary outcomes, safety, and follow-up details.

### 2.6 Risk of bias assessment

Two authors will evaluate the methodological study quality using Cochrane risk of bias tool. It includes aspects of selection bias, performance bias, detection bias, attrition bias, reporting bias, and other bias. Each aspect is further divided into as a low, unclear or high risk of bias. Any unresolved disagreements between 2 authors will be solved through discussion with another senior author.

### 2.7 Data synthesis

We will perform statistical analysis using RevMan 5.3 software. All dichotomous data will be expressed as risk ratio and 95% confidence intervals. All continuous data will be calculated as mean difference or standardized mean difference and 95% confidence intervals. The degree of statistical heterogeneity will be identified using *I*^*2*^ statistic. Acceptable heterogeneity is considered if *I*^*2*^ ≤50% and a fixed-effect model will be applied. Otherwise, obvious heterogeneity is regarded if *I*^*2*^ >50%, and a random-effect model will be utilized. If sufficient clinical and statistical data is collected on the same interventions, comparators, and outcomes, we will conduct meta-analysis when there is homogeneity among included studies. On the other hand, we will perform subgroup analysis or meta-regression to explore the possible reasons for the obvious heterogeneity. In addition, we will undertake a narrative description to synthesize data.

### 2.8 Subgroup analysis

We will carry out subgroup analysis or meta-regression based on the different interventions, controls and outcome measurements.

### 2.9 Sensitivity analysis

To test the robustness of the results data, we will perform sensitivity analysis by excluding studies with high risk of bias.

### 2.10 Reporting bias

When there are sufficient studies available, we will check the reporting bias using funnel plot and Egger regression test.^[21–22]^

### 2.11 Ethics and dissemination

No ethical approval is needed, because this study will not occupy individual patient data. We are expected to publish this study through a peer-reviewed journal.

## 3 Discussion

This study will synthesize the available direct evidence of the efficacy of rehabilitation used in the management of TCA in clinical practice. To our best knowledge, this is the first study considering treatment of TCA for the treatment of patients with OF. The results of this study have the potential influence on the management of TCA for patients with OF. The findings of this study will provide helpful evidence for the patients, clinician, as well as reference for the health-related policy maker.

## Author contributions

**Conceptualization:** Yan-Xiu Qi, Si-ying Li, Dong-lan Wang, Ping-ping Zhou.

**Data curation:** Dong-lan Wang.

**Formal analysis:** Si-ying Li, Dong-lan Wang, Ping-ping Zhou.

**Funding acquisition:** Ping-ping Zhou.

**Investigation:** Ping-ping Zhou.

**Methodology:** Yan-Xiu Qi, Si-ying Li, Dong-lan Wang.

**Project administration:** Ping-ping Zhou.

**Resources:** Yan-Xiu Qi, Si-ying Li, Dong-lan Wang.

**Software:** Yan-Xiu Qi, Si-ying Li, Dong-lan Wang.

**Supervision:** Ping-ping Zhou.

**Validation:** Yan-Xiu Qi, Dong-lan Wang, Ping-ping Zhou.

**Visualization:** Yan-Xiu Qi, Si-ying Li, Dong-lan Wang, Ping-ping Zhou.

**Writing – original draft:** Yan-Xiu Qi, Si-ying Li, Dong-lan Wang, Ping-ping Zhou.

**Writing – review & editing:** Yan-Xiu Qi, Si-ying Li, Ping-ping Zhou.
